# Trans-synaptic degeneration in the optic pathway. A study in clinically isolated syndrome and early relapsing-remitting multiple sclerosis with or without optic neuritis

**DOI:** 10.1371/journal.pone.0183957

**Published:** 2017-08-29

**Authors:** Marco Puthenparampil, Lisa Federle, Davide Poggiali, Silvia Miante, Alessio Signori, Elisabetta Pilotto, Francesca Rinaldi, Paola Perini, Maria Pia Sormani, Edoardo Midena, Paolo Gallo

**Affiliations:** 1 Multiple Sclerosis Centre, Department of Neurosciences DNS, University Hospital–Medical School, Padova, Italy; 2 Department of Health Sciences, Section of Biostatistics–University of Genova, Genova, Italy; 3 Ophthalmology Unit, Department of Neurosciences DNS, University Hospital–Medical School, Padova, Italy; Roskamp Institute, UNITED STATES

## Abstract

**Objective:**

Increasing evidence suggest that neuronal damage is an early and diffuse feature of Multiple Sclerosis (MS) pathology. Analysis of the optic pathway may help to clarify the mechanisms involved in grey matter damage in MS. Purpose of our study was to investigate the relationship between inflammation and neurodegeneration and to achieve evidence of trans-synaptic degeneration in the optic pathway in MS at clinical onset.

**Methods:**

50 clinically isolated syndromes/early relapse-onset MS (CIS/eRRMS) with mean disease duration of 4.0±3.5 months, 28 MRI healthy controls (HC) and 31 OCT-HC were studied. Ten patients had optic neuritis at presentation (MSON+), 40 presented with other symptoms (MSON-). MRI examination included 3D-T1, 3D-FLAIR and 3D-DIR sequences. Global cortical thickness (gCTh), pericalcarin CTh (pCTh) and white matter volume (WMV) were analysed by means of Freesurfer on 3D-T1 scans. Optic radiation morphology (OR) and volume (ORV) were reconstructed on the base of the Jülich’s Atlas. White matter lesion volume (WMLV), OR-WMLV and percent WM damage (WMLV/WMV = WMLV% and OR-WMLV/ORV = ORWMLV%) were obtained by 3D-FLAIR image segmentation. 3D-DIR sequences were applied to identify inflammatory lesions of the optic nerve. Optic coherence tomography (OCT) protocol included the analysis of global peripapillary retinal nerve fiber layer (g-RNFL) and the 6 *fundus oculi*’s sectors (temporal, T-RNFL; temporal superior, TS-RNFL; nasal superior, NS-RNFL; nasal, N-RNFL; nasal inferior, NI-RNFL, temporal inferior, TI-RNFL). The retina of both eyes was analyzed. The eyes of ON+ were further divided into affected (aON+) or not (naON+).

**Results:**

No difference in CTh was found between CIS/eRRMS and HC, and between MSON+ and MSON-. Moreover, MSON+ and MSON- did not differ for any WM lesion load parameter. The most significant correlations between RNFL thickness and optic radiation WM pathology were found in MSON+. In these patients, the temporal RNFL inversely correlated to ipsilateral optic radiation WM lesion load (T-RNFL: r -0.7, p<0.05; TS-RNFL: r -0.7, p<0.05), while nasal RNFL inversely correlated to contralateral optic radiation WM lesion load (NI: r -0.8, p<0.01; NS-RNFL: r -0.8, p<0.01).

**Conclusions:**

Our findings suggest that in MSON+ the optic pathway is site of a diffuse pathological process that involves both directly and via trans-synaptic degeneration the RNFL.

## Introduction

Increasing evidence suggests that grey matter (GM) is early and widely damaged in Multiple Sclerosis (MS). Histological and neuroimaging studies have disclosed variable degrees of inflammation [1–3] and diffuse neurodegeneration (atrophy) [4,5] in the cortex of MS patients, even at clinical onset and sometimes preceding the appearance of white matter (WM) lesions [6]. Cortical inflammation may be the cause of neuronal loss and GM atrophy. Nevertheless, retrograde or anterograde axonal degeneration, starting within WM lesions far from the cortex or in the normal appearing WM (NAWM), may also be responsible for neuronal loss and GM atrophy [7].

Due to its anatomical complexity, characterized by both WM and GM inter-connected structures, the optic pathway may offer the possibility of investigating the complex relationship between axonal damage in WM lesions and neuronal loss [8]. However, studies aimed at disclosing correlations between optical coherence tomography (OCT) and magnetic resonance imaging (MRI) findings have reported conflicting results. Indeed, the association between WM lesion volume (WMLV) in the optic radiation and retinal or cortical damage (suggested to be the expression of trans-synaptic degeneration) that was observed in several studies [7,9–13], was not be confirmed by Raz et al [14]. Several factors related to the methodology used, the patient populations analyzed (different disease duration and age) and the variable degrees of WM and GM lesion load in the optic pathway may explain literature discrepancies.

The predictive clinical values of any inflammatory or neurodegenerative parameter, as well as the possible relationship between WM and GM pathology, should be analyzed in the very early MS phases, possibly at clinical onset. Indeed, with MS progression the demonstration of any possible correlation between axonal damage in the WM, trans-synaptic degeneration and cortical atrophy is hampered by the widespread diffusion of both WM and GM pathology. Therefore, we analyzed the possible relationships between WM and GM pathology in the optic pathway in 50 patients with clinically isolated syndrome (CIS) or very early relapse-onset MS (eRRMS) in order to set the basis for a longitudinal study.

## Material and methods

### Study population

Fifty consecutive patients with a diagnosis of CIS suggestive of MS or eRRMS according to the most recent criteria [15] were included in the study. Diagnostic work-up included MRI (see below), Visual Evoked Potentials (VEP), detailed biochemical, immunological (including anti-extractable nuclear antibodies, anti-nuclear antibodies, Anti-neutrophil cytoplasmic antibodies, Angiotensin Converting Enzyme, anti-Phospholipid antibodies, anti-Aquaporin4 antibodies, Ab, anti-native DNA antibodies, anti-thyroid peroxidase antibodies, anti-thyroglobulin antibodies, anti-TSH Receptor antibodies, IgA-IgE-IgM-IgG concentration, C3, C4, circulating immunocomplex) and microbiological screenings in blood, and cerebrospinal fluid examination (i.e. albumin, IgG and glucose concentrations, cell count, IgG Index and IgG Hyperbolic Function, IsoElettroFocusing for detection of IgG Oligoclonal Bands (IgGOB); microbiological tests when necessary). Inclusion criteria were also disease duration < 12 months and age ≥18 years. Exclusion criteria included any systemic (including diabetes), ophthalmological (severe myopia: > -6.0 dp or axial eye length >26mm; severe hypermetropia: > 5 dp; cylinder >3 dp; optic disc drusen; cataracts; current or previous glaucoma; other causes of vision loss not attributable to MS) and drug-related causes of retinal damage. Patient’s OCT and MRI findings were compared to those of two independent cohort of 31 and 28 healthy controls (HC) respectively (hereafter abbreviated in HC-OCT and HC-MRI). Patients and HC did not differ for age and gender. The study was approved by the Ethics Committee of Azienda Ospedaliera di Padova and a written informed consent was obtained by all the participants.

### MRI protocol

Images were acquired using a 3Tesla MRI (Ingenia, Philips Medical Systems, Best, The Netherlands) with 33 mT/m power gradient and a 32-channel head coil. No major hardware upgrades occurred during the study, and bimonthly quality-assurance sessions assured measurement stability. The following images were acquired: (a) three-dimensional (3D) Fluid Attenuated Inversion Recovery (FLAIR): Repetition Time (RT) 4800 ms; Echo Time (ET) 310 ms; Inversion Time (IT) 1650 ms; 365 contiguous sagittal slices with thickness of 1.0 mm; matrix size 512 x 512; and FOV = 256 x 256 x 365 mm^3^; (b) 3D-turbo field echo T1 (TFE, 3D-T1): RT 7.8 ms; ET 3.6 ms; 180 contiguous sagittal slices with the off-centre positioned on zero with thickness of 1.0 mm; flip angle = 8°; matrix size = 220 x 220; and FOV = 220 x 220 x 180 mm^3^; (c)3D-Double Inversion Recovery (DIR) sequences: RT 5500 ms; ET 280 ms; 300 contiguous sagittal slices with the off-centre positioned on zero with thickness of 1.3 mm; flip angle = 90°; matrix size = 256 x 256; and FOV = 256 x 256 x 390 mm^3^; d) T2-weighted with combined fat and water suppression SPIR/FLAIR (8000/120 [TR/TE]; TI, 2200; field of view, 160mm2; matrix, 2562; signal average, 2; echo train length, 21; imaging time, 4 minutes) for the identification of high-signal lesions within the optic nerve. However, on the base of the results of two recent studies [16,17] that disclosed the superiority of 3D DIR sequence in detecting optic nerve lesions compared to FLAIR images, we used both sequences to depict optic nerve lesions. This was done by two independent observers (MP and DP) blinded to patient clinical data. All patients and HC were analysed twice to evaluate the intra-observer variability.

Both global cortical thickness (gCTh) and pericalcarin CTh (pCTh) were analysed by means of Freesurfer on 3D-T1 sequences. The WM volume (WMV) was also calculated by Freesurfer on 3D-T1 sequences, while the application of the Jülich probabilistic atlas (threshold at 0.2) to the same sequences allowed obtaining a binary masks of the optic radiations in the standard Montreal Neurological Institute space. This mask was then projected into the space of individual subjects to obtain the optic radiation volume (ORV). White matter lesions were identified in the space of individual subjects by consensus of three examiners (MP, DP, PG) and their volumes were calculated by a Python script. Optic radiation WMLV were obtained by the intersection of WM lesion mask and optic radiation mask. Furthermore, the WMLV/WMV ratio (WMLV%) and the optic radiation WMLV/ORV ratio (optic radiation WMLV%) were also calculated. Finally, in order to avoid the influence of global WMLV on optic radiation WMLV, the ratio between optic radiation WMLV% and global WMLV% was calculated (WMLV-ratio).

### OCT protocol

Spectral Domain retinal OCT (SPECTRALIS; Heidelberg Engineering, Carlsbad, CA; Heidelberg Eye Explorer version 1.7.0.0) examination of each eye was performed in all patients and controls by two observers (FL and PE). All the OCT scans fulfilled OSCAR-IB criteria [18,19]. OCT scans were acquired in a dark room, without the administration of any mydriatic agent. OCT scans included a peri-papillary 3.5 mm ring scan to measure retinal nerve fiber layer (RNFL) thickness, which was expressed both as global peri-papillary RNFL (g-RNFL) and as sectorial peri-papillary RNFL (temporal, T-RNFL; temporal superior, TS-RNFL; nasal superior, NS-RNFL; nasal, N-RNFL; nasal inferior, NI-RNFL); temporal inferior, TI-RNFL). The ring scan was manually superimposed to the optic nerve head. To exclude the presence of artifacts that interfered with RNFL segmentation and in order to confirm the automatic RNFL segmentation, an expert (FL or EP) reviewed all the OCT scans.

We analyzed the possible correlations between the temporal (T, TI, TS) and the nasal (N, NI, NS) fields of the RNFL respectively with the ipsilateral and the contralateral optic radiation WM pathology (WMLV and WMLV%), and between all RNFL sectors with global WMLV and WMLV%.

### Statistical analysis

Mean and standard deviation (SD) or median and interquartile range (IQR) were reported for continuous variables. Correlation between temporal, nasal and MRI values was calculated by mean of Pearson’s coefficient. To assess differences between groups for the clinical variables investigated and to highlight association between both temporal and nasal parameters and MRI characteristics (WMLV, WMLV%, ORWMLV, ORWMLV%, WMLV-ratio) a Generalized estimating equation (GEE) model was performed. Choice of GEE model was adopted to take in account the correlation between observations originated from the same patient (left and right eye). To statistically test whether correlation between temporal (or nasal) values and resonance magnetic parameters (MRI variables, namely WMLV (mm3), WMLV%, OR-WMLV (mm3), OR-WMLV%, OR-WMLV% / WMLV%, pericalcarinCTh and global Cth) was significantly different across the subgroups (MSON+ and MSON-), an interaction term between subgroups and MRI parameters was introduced into the GEE model. The unstructured correlation matrix was used as correlation structure for GEE model. The RNFL parameters were used as dependent variable in the GEE model.

Analysis of variance (ANOVA) was used to compare global CTh between groups (no different values within same patient). Since MRI values showed a strongly skewed distribution, a cube root transformation was adopted before the correlation analysis and GEE models. A p-value lower than 0.05 was considered statistically significant. Stata (v.13; StataCorp) has been used for the computation.

## Results

### Study population

No difference in the demographic features was observed among the groups of patients (Table 1, S1 Table). All patients presented a negative immunological and microbiological screening, while IgGOB were detected in 32 (64%) patients. Based on the clinical presentation, the patients were divided in two groups: 1) ‘optic neuritis’ MS onset (MSON+), this group was composed by 10 patients who had an acute or subacute monocular visual loss associated with retro-orbital pain during eye movement with an ophthalmological evaluation and a static perimetry confirming the diagnosis [20]; 2) ‘not optic neuritis’ MS onset (MSON-), 40 patients.

**Table 1:** Clinical and demographic parameters of all the subjects included in the study.

	Patients			Controls	
	*CIS/eRRMS*	*MSON+*	*MSON-*	*HC-OCT*	*HC-MRI*
***number***	50	10	40	31	28
***age (years)*** mean ± dev.st (range)	34.2±10.5 (18–59)	34.3±10.7 (22–56)	34.2±10.6 (18–59)	35.4±9.1 (25–59)	36.1±14.1 (18–66)
***female/male ratio***	1.8	1.5	1.9	1.4	3.7
***CIS suggestive of MS/eRRMS***	29/21	5/5	24/16	n.a	n.a.
***disease duration (months)*** mean ± dev.st	4.0 ± 3.5	4.5 ±4.1	3.9 ± 3.3	n.a.	n.a.
***EDSS*** median	1.5 (1–4)	1.25 (1–2.5)	2 (1–4)	n.a.	n.a.
***IgGOB*** (%)	32 (64%)	6 (60%)	26 (65%)	0 (0%)	0 (0%)
***delay onset-MRI (months)*** mean ± dev.st	4.0 ± 3.5	4.5 ±4.1	3.9 ± 3.3	n.a.	n.a.
***delay onset-OCT (months)*** mean ± dev.st	4.6 ± 3.5	5.8 ± 4.0	4.2 ± 3.3	n.a.	n.a.
***delay MRI-OCT (months)*** mean ± dev.st	0.64 ± 1.10	1.00 ± 0.94	0.55 ± 1.13	n.a.	n.a.

No significant difference was observed in any of the listed parameters between patient’s groups and controls, except for a mild difference between MRI-OCT delay between MSON- and MSON+. Abbreviations: Clinically Isolated Syndrome: CIS; early-Relapsing Remitting Multiple Sclerosis: eRRMS; Optic Neuritis patients: MSON+; Not Optic Neuritis patients: MSON-; MRI-Healty Control: HC-MRI; OCT-Healthy Control: HC-OCT; Expanded Disability Status Scale: EDSS; IgG Oligoclonal Bands: IgGOB; not applicable: n.a..

**Fig 1:**
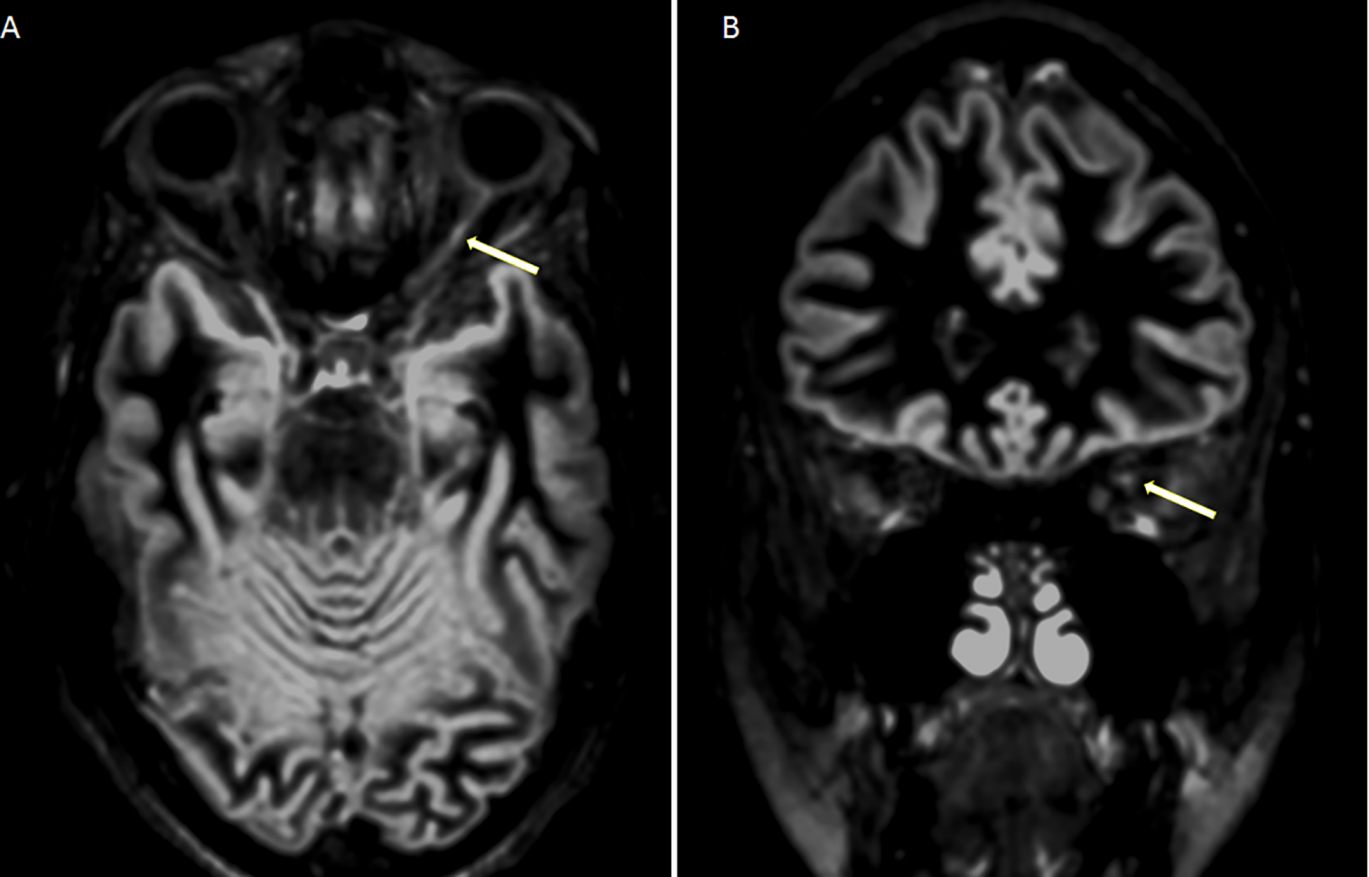
Double inversion recovery images (A Axial and B coronal) showing an inflammatory lesion (hyperintense signal) in the left optic nerve (arrows).

Interestingly, a clear-cut separation of the two groups was also achieved on the base of the MRI image of the optic nerve. Indeed, an inflammatory lesion in the affected optic nerve was observed on DIR images in all MSON+ patients by two independent evaluators blinded to clinical history, while no signal abnormality was found in the optic nerves of MSON- patients. The contribution of 3D-DIR in optic nerve lesion detection was confirmed to be particularly significant, with 100% accuracy for both operators and 0% intra-observer and inter-observer variability (Fig 1).

### White matter and cortical findings

WM lesions in the optic radiations were detected in 36/40 (90.0%) MSON- patients and 9/10 (90.0%) MSON+ patients (p = 0.99). No difference in WM pathology (i.e., global WMLV, optic radiation WMLV, global WMLV%, optic radiation WMLV%) was observed between the groups (Table 2).

**Table 2:** White matter MRI parameters in MSON+ and MSON-. Results are reported as median (IQR). P-values were obtained from GEE model. GEE analysis was performed at eye-level. White Matter Lesion Volume: WMLV; percentage of WMLV: WMLV%; optic radiation WMLV: OR-WMLV; percentage of OR-WMLV: OR-WMLV%. Other abbreviations as in Table 1.

	*MSON+*	*MSON-*	*p*
***WMLV (mm***^***3***^***)***	1369.3 (698.5–2816.2)	1721.4 (599.4–10716.7)	0.31
***WMLV%***	0.3 (0.1–0.6)	0.3 (0.1–2.3)	0.34
***OR-WMLV (mm***^***3***^***)***	74.3 (8.8–229.5)	72.3 (8.7–573.1)	0.24
***OR-WMLV%***	0.3 (0.03–0.9)	0.3 (0.03–2.0)	0.25
***OR-WMLV% / WMLV%***	0.87 (0.18–1.66)	0.88 (0.15–2.47)	0.97

Moreover, no difference between MSON+, MSON- and HC-MRI in both global and pericalcarin CTh was observed (p = 0.31 and p = 0.18 respectively, Table 3). Finally, no correlation was demonstrated between global or pericalcarin CTh and all metrics of WM pathology, age and gender.

**Table 3:** Cortical thickness values in MSON-, HC-MRI and MSON+.

Corticalareas		MSON- (mm)	HC-MRI (mm)	MSON+ (mm)	p-value
**Pericalcarin CTh**	(mean ± st.dev.)	1.70 ± 0.18	1.76 ± 0.20	1.66 (0.17)	0.28
**Global CTh**	(mean ± st.dev.)	2.48 ± 0.12	2.47 ± 0.11	2.40 ± 0.13	0.18

No difference was found among these three groups. Cortical thickness: CTh. Other abbreviations as in Table 1.

### RNFL

No difference in any OCT parameter was found between female and male subjects in each study group (HC-OCT, MSON+, MSON-) as well as no correlation between RNFL thickness and age was observed in patients and controls.

**Fig 2:**
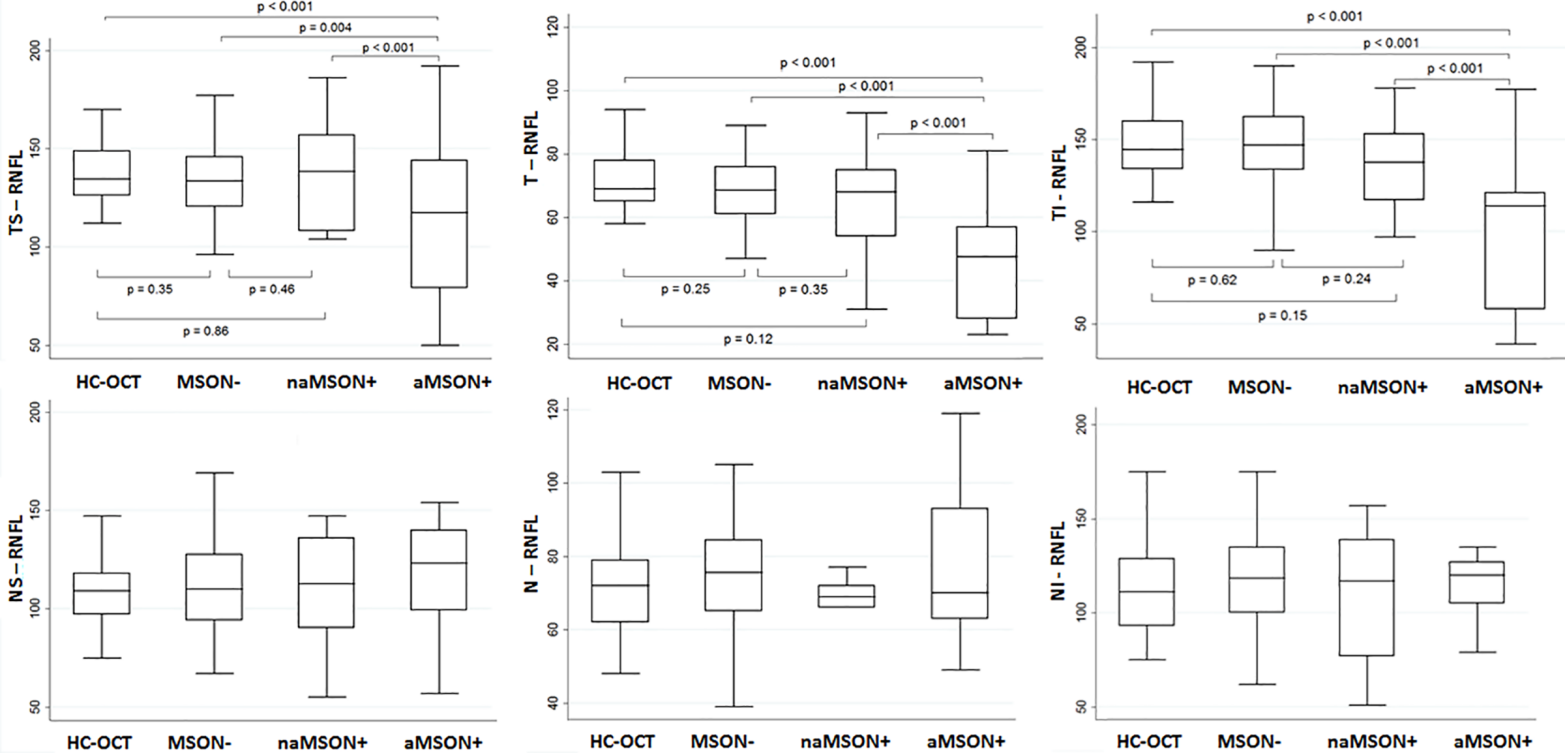
Comparison of the RNFL’s sector values. Healthy Controls: HC-OCT; Not Optic Neuritis MS patients: MSON-; Optic Neuritis MS patients: MSON+; affected eye MSON+: aON+; not affected eye MS ON+: naON+.

A thinner g-RNFL was observed in the affected eyes of MSON+ (83.3±25.3 μm) compared to both HC-OCT (99.6±9.3 μm, *p*<0.001) and MSON- (99.8±9.1 μm, *p*<0.001). Moreover, the affected eyes of MSON+ presented a significant thinning of T-RNFL, TI-RNFL and TS-RNFL (Fig 2, S2 Table) compared to their not affected eyes and to the eyes of HC-OCT and MSON-. Finally, the not affected eyes of both MSON+ and MSON- did not differ in RNFL thinning compared to the eyes of HC-OCT.

### Correlation between RNFL thickness and WM parameters

All the correlation data between RNFL findings and WM pathology parameters are reported in Table 4 (all raw data are enclosed as S3 Table). The most significant correlations were found between the temporal (TI and T-RNFL) sectors and both global and optic radiation WMLV and WMLV%. With regard to the nasal sectors, NI and NS mildly correlated with contralateral optic radiation WMLV and WMLV%.

**Table 4:** OCT and white matter pathology correlations.

	All observation (n = 100)					
	TI-RNFL	T-RNFL	TS-RNFL	NI-RNFL	N-RNFL	NS-RNFL
WMLV (mm^3^)	**-0.33 (0.001)**	**-0.25 (0.013)**	-0.19 (0.061)	**-0.21 (0.033)**	-0.07 (0.49)	-0.09 (0.36)
WMLV%	**-0.33 (0.001)**	**-0.24 (0.014)**	-0.19 (0.061)	**-0.23 (0.022)**	-0.09 (0.39)	-0.11 (0.29)
ORWMLV (mm3)	**-0.28 (0.006)**	**-0.20 (0.047)**	**-0.28 (0.005)**	**-0.23 (0.02)**	-0.14 (0.16)	**-0.25 (0.013)**
ORWMLV%	**-0.27 (0.006)**	-0.19 (0.055)	**-0.27 (0.007)**	**-0.24 (0.016)**	-0.15 (0.15)	**-0.24 (0.016)**
Ratio	-0.13 (0.20)	-0.05 (0.65)	**-0.25 (0.014)**	-0.19 (0.062)	-0.086 (0.40)	**-0.26 (0.001)**
	**MSON–(n = 80)**					
WMLV (mm^3^)	-0.16 (0.15)	-0.18 (0.11)	0 (0.99)	-0.15 (0.18)	-0.12 (0.27)	0.037 (0.75)
WMLV%	-0.15 (0.20)	-0.17 (0.14)	0.01 (0.97)	-0.18 (0.10)	-0.14 (0.20)	0.02 (0.87)
ORWMLV (mm^3^)	-0.14 (0.24)	-0.066 (0.56)	-0.16 (0.17)	-0.19 (0.084)	-0.12 (0.29)	-0.18 (0.11)
ORWMLV%	-0.13 (0.26)	-0.065 (0.57)	-0.15 (0.18)	-0.21 (0.066)	-0.13 (0.25)	-0.17 (0.12)
Ratio	-0.09 (0.43)	-0.004 (0.97)	-0.16 (0.17)	-0.14 (0.21)	-0.045 (0.69)	-0.20 (0.078)
	**MSON + not affected eyes (n = 10)**					
WMLV (mm^3^)	-0.15 (0.68)	0.01 (0.97)	-0.07 (0.85)	-0.16 (0.65)	0.08 (0.83)	-0.40 (0.26)
WMLV%	-0.15 (0.68)	0.01 (0.97)	-0.07 (0.85)	-0.16 (0.65)	0.08 (0.83)	-0.40 (0.26)
ORWMLV (mm^3^)	-0.52 (0.13)	-0.27 (0.46)	-0.43 (0.21)	-0.35 (0.33)	-0.07 (0.85)	-0.44 (0.20)
ORWMLV%	-0.58 (0.08)	-0.36 (0.30)	-0.50 (0.14)	-0.32 (0.36)	-0.04 (0.91)	-0.48 (0.16)
Ratio	-0.61 (0.059)	-0.46 (0.18)	**-0.73 (0.016)**	-0.31 (0.38)	-0.027 (0.94)	-0.53 (0.12)
	**MSON + affected eyes (n = 10)**					
WMLV (mm^3^)	-0.39 (0.26)	-0.55 (0.10)	-0.54 (0.11)	**-0.70 (0.025)**	-0.17 (0.64)	-0.50 (0.14)
WMLV%	-0.39 (0.26)	-0.55 (0.10)	-0.54 (0.11)	**-0.70 (0.025)**	-0.17 (0.64)	-0.50 (0.14)
ORWMLV (mm3)	-0.44 (0.20)	**-0.66 (0.037)**	**-0.67 (0.035)**	**-0.79 (0.006)**	-0.46 (0.18)	**-0.77 (0.009)**
ORWMLV%	-0.46 (0.19)	**-0.69 (0.029)**	**-0.64 (0.044)**	**-0.76 (0.011)**	-0.44 (0.20)	**-0.75 (0.013)**
Ratio	-0.38 (0.27)	-0.63 (0.053)	**-0.64 (0.044)**	-0.49 (0.15)	-0.48 (0.16)	**-0.66 (0.038)**

The temporal RNFL sectors were correlated with the ipsilateral OR, while the nasal sectors were correlated with the contralateral OR. P-values reported for “All observations” and MSON- groups derived from GEE, which was applied in order to consider inter-eye correlation. Since within MSON+ affected and not affected eyes all observations were from independent patients, Pearson correlation was performed.

Retinal nerve fibre layer: RNFL; global RNFL: g-RNFL; temporal inferior RNFL: TI-RNFL; temporal RNFL: T-RNFL; temporal superior RNFL, TS-RNFL; nasal superior RNFL: NS-RNFL; nasal RNFL: N-RNFL; nasal inferior RNFL: NI-RNFL Other abbreviations as in Tables 1 and 2.

### Stratified analysis by history of optic neuritis

The interaction analysis disclosed that lower values of TI, T and TS-RNFL associated with higher values of optic radiation WMLV (p<0.01, p<0.05 and p<0.05, respectively) and WMLV% (p < 0.005; p < 0.05 and p< 0.005, respectively) in the affected eye of MSON+ compared to MSON- eyes. Similarly, lower values of TS-RNFL (p<0.05) were associated to higher values of global WMLV in MSON+ affected eyes compared with MSON-.

In MSON- the correlation between RNFL and WM parameters was not confirmed. In MSON+ the temporal RNFL inversely correlated to ipsilateral optic radiation WM lesion load (T-RNFL: r -0.7, p<0.05; TS-RNFL: r -0.7, p<0.05), while nasal RNFL inversely correlated to contralateral optic radiation WM lesion load (NI: r -0.8, p<0.01; NS-RNFL: r -0.8, p<0.01).

### Correlation between RNFL thickness and cortical thickness

No correlation between RNFL and CTh was observed in both affected and not affected eyes of MSON+, while in MSON- mild correlations were disclosed between gCTh and T-RNFL (r:-0.28, p<0.05), TS-RNFL (R:-0.35, p<0.005) and N-RNFL (r:0.23, p<0.05).

When applying the GEE analysis, the correlation between gCTh and TI-RNFL differed within groups and reached the statistical significance (p<0.05). The correlation was positive in the affected eye of MSON+ (r:0.55, p = 0.10), but was negative in MSON- (r: -0.15, p = 0.2). The correlation between gCTh and T-RNFL differed among the three groups with a trend toward the significance, that was not reached (p = 0.064).

When considering pCTh, the correlation with T-RNFL (Table 5) was positive when considering the affected eye of MSON+ (r: 0.43, p = 0.2) but negative in the other subgroups (i.e., r:-0.19, p = 0.1 in MSON- and r:-0.44, p = 0.20 in the MSON+ not affected eye). When applying GEE analysis, the difference among subgroups reached the significance (p<0.05). Finally, a trend in the correlation between TS-RNFL and pCTh (p = 0.054) was observed.

**Table 5:** Correlations between RNFL and cortical thickness.

	All observation (n = 100)					
	TI-RNFL	T-RNFL	TS-RNFL	NI-RNFL	N-RNFL	NS-RNFL
**pCTh**	0.12 (0.25)	-0.09 (0.36)	-0.06 (0.52)	0.13 (0.20)	0.08 (0.45)	0 (0.99)
**gCTh**	0.13 (0.21)	-0.03 (0.79)	-0.14 (0.18)	0.05 (0.61)	**0.23 (0.024)**	0.0 (0.50)
	**MSON- (n = 80)**					
**pCTh**	0.01 (0.90)	-0.19 (0.084)	-0.16 (0.17)	0.11 (0.32)	0.09 (0.42)	0.04 (0.70)
**gCTh**	-0.15 (0.17)	**-0.28 (0.011)**	**-0.35 (0.002)**	0.06 (0.62)	**0.26 (0.019)**	0.05 (0.69)
	**MSON+ not affected eyes (n = 10)**					
**pCTh**	0.03 (0.93)	-0.44 (0.20)	-0.47 (0.17)	0.31 (0.39)	0.25 (0.48)	0.19 (0.60)
**gCTh**	0.02 (0.95)	0.13 (0.72)	0.01 (0.99)	0.16 (0.67)	0.14 (0.70)	0.24 (0.51)
	**MSON+ affected eyes (n = 10)**					
**pCTh**	0.45 (0.19)	0.43 (0.21)	0.42 (0.23)	0.02 (0.96)	-0.21 (0.57)	-0.45 (0.19)
**gCTh**	0.55 (0.10)	0.27 (0.45)	0.19 (0.60)	-0.19 (0.60)	-0.01 (0.99)	0.19 (0.60)

The analysis was performed correlating the temporal RNFL sectors with the ipsilateral pericalcarin CTh (pCTh) and the nasal RNFL sectors with the contralateral pCTh. P-values reported for “All observations” and MSON- groups derived from GEE, which was applied in order to consider inter-eye correlation. Since within MSON+ affected and not affected eyes all observations were from independent patients, Pearson correlation was performed. All the sectors were correlated to global CTh (gCTh). Other abbreviations as in Tables 1, 2 and 4.

## Discussion

Several pathological processes may contribute to determine neurodegeneration in MS, such as GM inflammation, retrograde axonal degeneration and trans-synaptic degeneration. Indeed, variable degrees of meningeal and cortical inflammation have been described in all the clinical types of MS [21,22] and locally produced neurotoxins and pro-apoptotic signals have been proposed to play a major role in determining neuronal loss [23–26]. Axonal damage in WM lesions and in NAWM far from the cortex may also play a relevant role in neurodegeneration: an extensive axonal transection has been demonstrated in active WM lesions [27], black holes (areas of severe axonal loss) are commonly observed in T1 weighted MRI scans [28] and anterograde and/or retrograde trans-synaptic degenerations have also been suggested to contribute to neuronal loss and GM atrophy [29–32]. However, the demonstration of any possible correlation between WM damage, trans-synaptic degeneration and cortical atrophy in MS is hampered by the progressive and widespread diffusion of both WM and GM pathology with disease progression. Thus, we studied CIS highly suggestive of MS and eRRMS, with very short disease duration, not treated with disease modifying therapies and having no co-morbidity, and found that in MSON+ the optic pathway is likely site of a diffuse pathological process involving the RNFL either directly or via trans-synaptic degeneration.

We observed a lack of correlation between WM (global or optic radiation WM lesion load) and GM (global or pericalcarin CTh) damage. This finding questions a role for anterograde trans-synaptic degeneration in determining cortical atrophy in very early clinical MS phases. However, anterograde trans-synaptic degeneration may become more evident in later disease phases, as suggested by observations in patients with longer disease duration (i.e., 9.0 years), where a correlation between optic radiation WM lesion load and cortical volume could be observed [7].

In our study the role played by axonal damage in the NAWM, a matter of controversial debate [33], was not investigated. Recently, cortical atrophy was demonstrated to associate with a loss of NAWM integrity in MS patients with long-standing disease [34]. However, combined histopathology and MRI studies have suggested that while both demyelination and axonal damage are present in lesions, the NAWM damage particularly consists of subtle demyelination [35]. Moreover, NAWM damage was observed to increase with disease progression. Thus, we believe that, given the very short mean disease duration (4.0±3.5 months) of our cohort of CIS/eRRMS, the eventual role played by axonal damage in NAWM should be considered negligible.

The most interesting finding of our study was the observation of an inverse association between RNFL thickness and WM lesion load in the optic radiation (ipsilateral for temporal RNFL and contralateral for nasal RNFL) only in MSON+ patients. This finding seems to indicate that initial mechanism(s) of retrograde degeneration, which proceeds from retro-chiasmatic structures to RNFL, may occurs in these patients. It might be possible that the combination of optic neuritis and WM damage in the optic pathway triggers trans-synaptic degenerations. The involvement of the Lateral Geniculate Nucleus (LGN) in this process is suggested by the observation of a significant neural loss in this nucleus [29], especially in the parvocellular neuron layers [36]. However, these findings could only partially be explained by retrograde degeneration, since a damage of the optic radiation accounts for a minor percentage (14–28%) of LGN volume loss [29]. Moreover, the loss of parvocellular neurons in LGN was found to correlate with ON damage [36], thus indicating an anterograde trans-synaptic degeneration involving this nucleus and then spreading to the optic radiation, as demonstrated by connectivity changes in the optic radiation of MS patients with a previous history of optic neuritis [37].

No clear evidence of trans-synaptic (retrograde or anterograde) degeneration could be found in MSON-. Indeed, in these patients RNFL thickness did not correlate with optic radiation WMLV or pericalclarin CTh and pericalcarin CTh did not associate to optic radiation WMLV. Since the correlation between T-RNFL and gCTh did not reach the significance in all MS subgroups, the difference in affected and not-affected eye MSON+ and MSON- disclosed by GEE has be taken with caution and eventually confirmed in a larger cohort of patients. Since MSON- and MSON+ did not differ in optic radiation or global WMLV, the role played by optic neuritis seems to be pivotal within the complex pathological process occurring in the optic pathway of ON+ MS patients.

We are aware of the limitations of our study. First, the low number of MSON+ patients, that, however, is in line with the percentage of optic neuritis observed at MS onset (20–25%). Despite this limitation, the associations between RNFL thickness and WM lesion load in the optic radiation in these patients reached the statistical significance. A study on a larger cohort of MSON+ is currently in progress. Second, the lack of other OCT parameters. We point out that our study was designed to evaluate the RNFL as a possible site of neurodegeneration. Further information might rise from the analysis of macular volume and ganglion cell layer thickness, that were not included in the present study. Third, multi-focal electroretinogram and/or multi-focal visual evoked potential may help to understand the functional damage associated to neurodegeneration.

In summary, in ON+ patients the optic nerve inflammation co-operate with optic radiation WM lesion load in determining a more diffuse damage in the optic pathway, that leads to RNFL thinning and LGN neuronal loss. Trans-synaptic degeneration may be one of the mechanisms that links inflammation and neurodegeneration at least in these patients.

## Supporting information

S1 Table Clinical and demographical features of CIS/eRRMS patients. (PDF)

S2 Table Global and sectorial RNFL values in the 4 groups of patients. Only temporal field RNFL (TI-RNFL, T-RNFL and TS-RNFL) was reduced in affected eye of MSON+ when compared to HC, MSON- and not affected eye of ON+. No difference were found for the nasal field (NI-RNFL, N-RNFL, NS-RNFL). OCT healthy controls: HC-OCT; MS patients without previous medical history of optic neuritis: MSON-; MS patients with medical history of optic neuritis: MSON+; retinal nerve fibre layer: RNFL; global RNFL: g-RNFL; temporal inferior RNFL: TI-RNFL; temporal RNFL: T-RNFL; temporal superior RNFL, TS-RNFL; nasal superior RNFL: NS-RNFL; nasal RNFL: N-RNFL; nasal inferior RNFL: NI-RNFL. *: p < 0.005 when compared to affected MSON+; **: p < 0.001 when compared to affected MSON+.
(PDF)

S3 Table OCT and MRI parameters of the study population. (PDF)
