# Supplementary material for: Trans-synaptic degeneration in the optic pathway. A study in clinically isolated syndrome and early relapsing-remitting multiple sclerosis with or without optic neuritis
Source: PLoS One. 2017 Aug 29;12(8):e0183957. doi: 10.1371/journal.pone.0183957 (PMC5574611; doi:10.1371/journal.pone.0183957)
Supplement: S1 Table — (PDF) [file pone.0183957.s001.pdf]

S1 Table. Clinical and demographical features of CIS/eRRMS patients.

Code	Gender (1F)	disease onset	Lumbar Punture		MRI				OCT			Visual Evoked Potentials			Diagnosis	Eye of ON
			date	IgGOB	date	EDSS	age	dT onset	date	dT onset	dT MRI	date	dT onset	result		
01-CIS/eRRMS	1	29/12/2014	26/01/2015	IgGOB+	12/02/2015	1,5	37	1,00	26/01/2015	0,00	0,00	26/01/2015	0,00	normal	Relapsing-Remitting MS	0
02-CIS/eRRMS	1	01/08/2014	24/02/2015	IgGOB-	17/03/2015	1,5	50	7,00	17/03/2015	7,00	0,00	24/02/2015	6,00	normal	CIS suggestive of MS	0
03-CIS/eRRMS	1	30/12/2014	27/01/2015	IgGOB+	26/01/2015	2,0	47	0,00	30/01/2015	1,00	0,00	27/01/2015	0,00	normal	CIS suggestive of MS	0
04-CIS/eRRMS	1	18/10/2014	21/11/2014	IgGOB+	24/11/2014	2,0	30	1,00	04/03/2015	4,00	3,00	26/11/2014	1,00	normal	Relapsing-Remitting MS	0
05-CIS/eRRMS	0	15/06/2014	22/08/2014	IgGOB+	26/08/2014	1,0	47	2,00	04/02/2015	7,00	5,00	25/08/2014	2,00	normal	Relapsing-Remitting MS	0
06-CIS/eRRMS	1	28/02/2015	07/03/2015	IgGOB+	11/03/2015	2,0	24	0,00	10/03/2015	0,00	0,00	10/03/2015	0,00	normal	CIS suggestive of MS	0
07-CIS/eRRMS	0	01/10/2014	12/02/2015	IgGOB+	10/03/2015	3,5	24	5,00	12/02/2015	4,00	0,00	10/03/2015	5,00	normal	Relapsing-Remitting MS	0
08-CIS/eRRMS	1	01/07/2014	13/01/2015	IgGOB-	20/01/2015	4,0	35	6,00	09/12/2014	5,00	1,00	13/01/2015	6,00	normal	CIS suggestive of MS	0
09-CIS/eRRMS	0	02/11/2014	07/11/2014	IgGOB-	21/01/2015	3,0	44	2,00	21/01/2015	2,00	0,00	18/11/2014	0,00	normal	CIS suggestive of MS	0
10-CIS/eRRMS	0	01/10/2014	11/11/2014	IgGOB+	18/12/2014	3,0	26	2,00	18/12/2014	2,00	0,00	12/11/2014	1,00	normal	CIS suggestive of MS	0
11-CIS/eRRMS	0	20/05/2014	24/07/2014	IgGOB+	12/08/2014	2,0	27	2,00	12/08/2014	2,00	0,00	07/08/2014	2,00	normal	Relapsing-Remitting MS	0
12-CIS/eRRMS	1	01/04/2014	28/07/2014	IgGOB+	13/08/2014	1,0	33	4,00	13/08/2014	4,00	0,00	28/07/2014	3,00	normal	CIS suggestive of MS	0
13-CIS/eRRMS	0	01/07/2014	23/01/2015	IgGOB-	05/02/2015	2,5	21	7,00	05/02/2015	7,00	0,00	05/02/2015	7,00	normal	CIS suggestive of MS	0
14-CIS/eRRMS	0	01/11/2013	11/03/2014	IgGOB+	10/04/2014	1,0	44	5,00	19/04/2014	5,00	0,00	10/04/2014	5,00	normal	CIS suggestive of MS	0
15-CIS/eRRMS	0	01/12/2013	13/03/2014	IgGOB-	26/03/2014	1,0	30	3,00	19/04/2014	4,00	0,00	26/03/2014	3,00	normal	Relapsing-Remitting MS	0
16-CIS/eRRMS	0	01/09/2014	26/09/2014	IgGOB+	26/09/2014	2,0	26	0,00	17/10/2014	1,00	0,00	29/09/2014	0,00	normal	Relapsing-Remitting MS	0
17-CIS/eRRMS	0	01/11/2014	02/02/2015	IgGOB-	17/02/2015	2,0	46	3,00	17/02/2015	3,00	0,00	02/02/2015	3,00	normal	Relapsing-Remitting MS	0
18-CIS/eRRMS	1	20/10/2014	27/10/2014	IgGOB+	22/01/2015	2,0	27	3,00	22/01/2015	3,00	0,00	21/10/2014	0,00	normal	CIS suggestive of MS	0
19-CIS/eRRMS	1	08/01/2015	15/01/2015	IgGOB-	14/01/2015	3,5	22	0,00	04/02/2015	0,00	0,00	12/01/2015	0,00	normal	Relapsing-Remitting MS	0
20-CIS/eRRMS	1	20/07/2014	26/08/2014	IgGOB+	20/08/2014	2,0	28	1,00	26/08/2014	1,00	0,00	26/08/2014	1,00	normal	Relapsing-Remitting MS	0
21-CIS/eRRMS	1	30/03/2014	29/04/2014	IgGOB+	28/05/2014	1,0	39	1,00	12/07/2014	3,00	1,00	22/05/2014	1,00	normal	Relapsing-Remitting MS	0
22-CIS/eRRMS	0	01/02/2014	06/05/2014	IgGOB+	29/05/2014	1,0	42	3,00	12/07/2014	5,00	1,00	29/05/2014	3,00	normal	Relapsing-Remitting MS	0
23-CIS/eRRMS	0	01/08/2013	03/03/2014	IgGOB+	03/04/2014	2,0	18	8,00	21/05/2014	9,00	1,00	03/04/2014	8,00	normal	Relapsing-Remitting MS	0
24-CIS/eRRMS	1	01/03/2014	26/05/2014	IgGOB-	08/05/2014	2,0	32	2,00	07/08/2014	5,00	2,00	26/05/2014	2,00	normal	CIS suggestive of MS	0
25-CIS/eRRMS	1	01/11/2013	04/04/2014	IgGOB+	07/05/2014	1,0	34	6,00	18/08/2014	9,00	3,00	05/05/2014	6,00	normal	CIS suggestive of MS	0
26-CIS/eRRMS	1	05/01/2014	29/09/2014	IgGOB+	16/10/2014	2,5	46	9,00	29/09/2014	8,00	0,00	29/09/2014	8,00	normal	Relapsing-Remitting MS	0
27-CIS/eRRMS	1	01/05/2014	22/07/2014	IgGOB+	14/10/2014	2,0	42	5,00	26/08/2014	3,00	1,00	22/07/2014	2,00	normal	Relapsing-Remitting MS	0
28-CIS/eRRMS	1	01/04/2014	14/07/2014	IgGOB-	01/10/2014	2,0	59	6,00	04/09/2014	5,00	0,00	22/05/2014	1,00	normal	CIS suggestive of MS	0
29-CIS/eRRMS	1	10/06/2014	10/06/2014	IgGOB+	14/08/2014	1,0	23	2,00	18/06/2014	0,00	1,00	17/06/2014	0,00	normal	CIS suggestive of MS	0
30-CIS/eRRMS	1	14/05/2014	14/05/2014	IgGOB+	30/09/2014	1,5	49	4,00	17/06/2014	1,00	3,00	30/09/2014	4,00	normal	CIS suggestive of MS	0
31-CIS/eRRMS	0	01/09/2014	26/02/2015	IgGOB+	24/03/2015	2,0	22	6,00	19/03/2015	6,00	0,00	27/02/2015	5,00	normal	CIS suggestive of MS	0
32-CIS/eRRMS	0	01/12/2014	01/02/2015	IgGOB+	19/05/2015	1,5	43	5,00	19/05/2015	5,00	0,00	18/02/2015	2,00	normal	CIS suggestive of MS	0
33-CIS/eRRMS	1	01/01/2015	11/05/2015	IgGOB-	04/06/2015	1,0	23	5,00	04/06/2015	5,00	0,00	11/05/2015	4,00	normal	CIS suggestive of MS	0
34-CIS/eRRMS	1	01/04/2015	19/05/2015	IgGOB-	03/06/2015	1,0	41	2,00	03/06/2015	2,00	0,00	19/05/2015	1,00	normal	CIS suggestive of MS	0
35-CIS/eRRMS	1	07/03/2015	31/03/2015	IgGOB+	11/03/2015	3,0	18	0,00	02/04/2015	0,00	0,00	31/03/2015	0,00	normal	CIS suggestive of MS	0
36-CIS/eRRMS	1	01/11/2014	13/05/2015	IgGOB+	27/05/2015	1,0	22	6,00	28/05/2015	6,00	0,00	13/05/2015	6,00	normal	CIS suggestive of MS	0
37-CIS/eRRMS	1	01/04/2015	28/04/2015	IgGOB+	28/04/2015	3,0	25	0,00	06/05/2015	1,00	0,00	09/04/2015	0,00	normal	Relapsing-Remitting MS	0
38-CIS/eRRMS	1	01/12/2014	21/04/2015	IgGOB-	29/04/2015	1,0	34	4,00	29/04/2015	4,00	0,00	21/04/2015	4,00	normal	CIS suggestive of MS	0
39-CIS/eRRMS	1	09/05/2014	05/07/2015	IgGOB-	20/05/2015	1,0	46	12,00	20/05/2015	12,00	0,00	05/05/2015	11,00	normal	CIS suggestive of MS	0
40-CIS/eRRMS	1	01/01/2014	31/03/2015	IgGOB-	28/04/2015	1,0	41	15,00	28/04/2015	15,00	0,00	28/04/2015	15,00	normal	CIS suggestive of MS	0
41-CIS/eRRMS	1	01/07/2014	01/07/2014	IgGOB+	15/01/2015	1,5	34	6,00	17/01/2015	6,00	0,00	17/12/2014	5,00		Relapsing-Remitting MS	RE

S1 Table. Clinical and demographical features of CIS/eRRMS patients *(continue)*.

Code	Gender (1F)	disease onset	Lumbar Punture		MRI				OCT			Visual Evoked Potentials			Diagnosis	Eye of ON
			date	IgGOB	date	EDSS	age	dT onset	date	dT onset	dT MRI	date	dT onset	result		
42-CIS/eRRMS	1	01/06/2013	20/02/2014	IgGOB+	01/04/2014	1,0	38	10,00	26/06/2014	12,00	2,00	13/02/2014	8,00		Relapsing-Remitting MS	RE
43-CIS/eRRMS	0	01/04/2013	31/01/2014	IgGOB-	11/02/2014	2,5	56	10,00	23/02/2014	10,00	0,00	11/02/2014	10,00		Relapsing-Remitting MS	RE
44-CIS/eRRMS	0	03/04/2014	12/05/2014	IgGOB-	23/04/2014	1,0	41	0,00	07/07/2014	3,00	2,00	22/04/2014	0,00		CIS suggestive of MS	RE
45-CIS/eRRMS	1	05/04/2014	13/05/2014	IgGOB-	17/05/2014	1,5	22	1,00	30/07/2014	3,00	2,00	10/06/2014	2,00		Relapsing-Remitting MS	LE
46-CIS/eRRMS	1	01/05/2014	17/07/2014	IgGOB+	30/07/2014	1,0	29	2,00	27/08/2014	3,00	0,00	30/07/2014	2,00		CIS suggestive of MS	LE
47-CIS/eRRMS	1	08/06/2013	18/02/2014	IgGOB+	08/04/2014	1,0	44	10,00	24/06/2014	12,00	2,00	18/02/2014	8,00		Relapsing-Remitting MS	LE
48-CIS/eRRMS	0	01/03/2014	16/05/2014	IgGOB+	11/06/2014	1,5	23	3,00	25/06/2014	3,00	0,00	16/05/2014	2,00		CIS suggestive of MS	LE
49-CIS/eRRMS	1	01/05/2014	27/05/2014	IgGOB+	18/06/2014	1,0	25	1,00	09/08/2014	3,00	1,00	27/05/2014	0,00		CIS suggestive of MS	LE
50-CIS/eRRMS	0	01/12/2013	14/01/2014	IgGOB-	12/02/2014	1,5	31	2,00	23/03/2014	3,00	1,00	16/12/2013	0,00		CIS suggestive of MS	LE
