# Supplementary material for: Trans-synaptic degeneration in the optic pathway. A study in clinically isolated syndrome and early relapsing-remitting multiple sclerosis with or without optic neuritis
Source: PLoS One. 2017 Aug 29;12(8):e0183957. doi: 10.1371/journal.pone.0183957 (PMC5574611; doi:10.1371/journal.pone.0183957)
Supplement: S2 Table — Only temporal field RNFL (TI-RNFL, T-RNFL and TS-RNFL) was reduced in affected eye of MSON+ when compared to HC, MSON- and not affected eye of ON+. No difference were found for the nasal field (NI-RNFL, N-RNFL, NS-RNFL). OCT healthy controls: HC-OCT; MS patients without previous medical history of optic neuritis: MSON-; MS patients with medical history of optic neuritis: MSON+; retinal nerve fibre layer: RNFL; global RNFL: g-RNFL; temporal inferior RNFL: TI-RNFL; temporal RNFL: T-RNFL; temporal superior RNFL, TS-RNFL; nasal superior RNFL: NS-RNFL; nasal RNFL: N-RNFL; nasal inferior RNFL: NI-RNFL. *: p < 0.005 when compared to affected MSON+; **: p < 0.001 when compared to affected MSON+. (PDF) [file pone.0183957.s002.pdf]

S2 Table. Global and sectorial RNFL values in the 4 groups of patients. Only temporal field RNFL (TI-RNFL, T-RNFL and TS-RNFL) was reduced in affected eye of MSON+ when compared to HC, MSON- and not affected eye of ON+. No difference were found for the nasal field (NI-RNFL, N-RNFL, NS-RNFL).

OCT healthy controls: HC-OCT; MS patients without previous medical history of optic neuritis: MSON-; MS patients with medical history of optic neuritis: MSON+; retinal nerve fibre layer: RNFL; global RNFL: g-RNFL; temporal inferior RNFL: TI-RNFL; temporal RNFL: T-RNFL; temporal superior RNFL, TS-RNFL; nasal superior RNFL: NS-RNFL; nasal RNFL: N-RNFL; nasal inferior RNFL: NI-RNFL. *: $p < 0.005$ when compared to affected MSON+; **: $p < 0.001$ when compared to affected MSON+.

	**HC-OCT (62 eyes)**	**MSON- (80 eyes)**	**MSON+**	
			**(10 not affected-eyes)**	**(10 affected-eyes)**
**g-RNFL**	99.6 ± 9.3**	99.8 ± 9.1**	99.7 ± 15.6	83.3 ± 25.3
**TI-RNFL**	148.9 ± 20.1**	146.6 ± 11.3**	137.5 ± 25.1**	105.3 ± 43.4
**T-RNFL**	72.2 ± 9.5**	69.6 ± 11.3**	65.9 ± 18.4**	46.4 ± 18.2
**TS-RNFL**	138.2 ± 14.5*	134.5 ± 17.6*	139.4 ± 28.2**	115.4 ± 43.4
**NI-RNFL**	112.9 ± 23.5	117.5 ± 27.1	110.7 ± 35.8	118.6 ± 28.5
**N-RNFL**	72.1 ± 13.5	75.4± 14.8	66.7 ± 16.0	76.2 ± 21.6
**NS-RNFL**	109.0 ± 16.9	109.7 ± 22.7	109.3 ± 31.9	118.1 ± 28.1
