# Supplementary material for: Trans-synaptic degeneration in the optic pathway. A study in clinically isolated syndrome and early relapsing-remitting multiple sclerosis with or without optic neuritis
Source: PLoS One. 2017 Aug 29;12(8):e0183957. doi: 10.1371/journal.pone.0183957 (PMC5574611; doi:10.1371/journal.pone.0183957)
Supplement: S3 Table — (PDF) [file pone.0183957.s003.pdf]

S3 Table. OCT and MRI parameters of the study population.

group	Code	OR	gender	age	ON (1= yes)	LE	RE	T-ipsilat.			N-controlat			WM			ipsilateral (to T-RNFL) ORWM				CTh	
						Gsn	Gdx	TI	T	TS	NI	N	NS	WMLV	WMV	%WMLV	OR WMLV mm^3	ORV	ORWMLV fraction (%)	ratio/ ratio	pericalcarine	global
hCOCT	01-HCOCT	dx	*M*	*29*	0	90	94	131	76	159	84	53	113									
hCOCT	01-HCOCT	sn	*M*	*29*	0	90	94	138	68	142	93	54	111									
hCOCT	02-HCOCT	dx	*F*	*58*	0	112	107	173	91	166	149	70	117									
hCOCT	02-HCOCT	sn	*F*	*58*	0	112	107	167	87	149	111	59	104									
hCOCT	03-HCOCT	dx	*M*	*39*	0	110	107	154	70	154	133	92	115									
hCOCT	03-HCOCT	sn	*M*	*39*	0	110	107	155	65	163	121	87	111									
hCOCT	04-HCOCT	dx	*M*	*34*	0	102	104	158	74	150	117	78	114									
hCOCT	04-HCOCT	sn	*M*	*34*	0	102	104	153	68	141	119	77	105									
hCOCT	05-HCOCT	dx	*F*	*32*	0	112	112	211	91	125	150	74	93									
hCOCT	05-HCOCT	sn	*F*	*32*	0	112	112	192	93	130	143	75	83									
hCOCT	06-HCOCT	dx	*M*	*30*	0	90	96	149	74	143	96	64	109									
hCOCT	06-HCOCT	sn	*M*	*30*	0	90	96	128	62	131	91	65	106									
hCOCT	07-HCOCT	dx	*M*	*33*	0	100	85	124	63	126	142	103	91									
hCOCT	07-HCOCT	sn	*M*	*33*	0	100	85	123	65	112	95	63	84									
hCOCT	08-HCOCT	dx	*F*	*30*	0	107	113	154	70	160	121	77	125									
hCOCT	08-HCOCT	sn	*F*	*30*	0	107	113	163	72	152	134	93	131									
hCOCT	09-HCOCT	dx	*F*	*31*	0	105	106	160	69	134	135	82	129									
hCOCT	09-HCOCT	sn	*F*	*31*	0	105	106	143	66	135	143	89	109									
hCOCT	10-HCOCT	dx	*F*	*32*	0	96	94	135	68	142	111	76	123									
hCOCT	10-HCOCT	sn	*F*	*32*	0	96	94	123	61	138	98	69	103									

S3 Table. OCT and MRI parameters of the study population *(continue)*.

group	Code	OR	gender	age	ON (1= yes)	LE	RE	T-ipsilat.			N-controlat			WM			ipsilateral (to T-RNFL) ORWM				CTh	
						Gsn	Gdx	TI	T	TS	NI	N	NS	WMLV	WMV	%WMLV	OR WMLV mm^3	ORV	ORWMLV fraction (%)	ratio/ ratio	pericalcarine	global
hCOCT	11-HCOCT	dx	*F*	*30*	0	95	97	159	81	140	78	78	102	[redacted]	[redacted]	[redacted]	[redacted]	[redacted]	[redacted]	[redacted]	[redacted]	[redacted]
hCOCT	11-HCOCT	sn	*F*	*30*	0	95	97	139	68	149	75	75	90	[redacted]	[redacted]	[redacted]	[redacted]	[redacted]	[redacted]	[redacted]	[redacted]	[redacted]
hCOCT	12-HCOCT	dx	*F*	*44*	0	88	86	132	62	126	96	60	115	[redacted]	[redacted]	[redacted]	[redacted]	[redacted]	[redacted]	[redacted]	[redacted]	[redacted]
hCOCT	12-HCOCT	sn	*F*	*44*	0	88	86	123	63	127	102	56	93	[redacted]	[redacted]	[redacted]	[redacted]	[redacted]	[redacted]	[redacted]	[redacted]	[redacted]
hCOCT	13-HCOCT	dx	*F*	*42*	0	97	91	128	58	124	126	73	125	[redacted]	[redacted]	[redacted]	[redacted]	[redacted]	[redacted]	[redacted]	[redacted]	[redacted]
hCOCT	13-HCOCT	sn	*F*	*42*	0	97	91	143	58	119	117	77	89	[redacted]	[redacted]	[redacted]	[redacted]	[redacted]	[redacted]	[redacted]	[redacted]	[redacted]
hCOCT	14-HCOCT	dx	*M*	*30*	0	91	93	143	78	141	104	62	106	[redacted]	[redacted]	[redacted]	[redacted]	[redacted]	[redacted]	[redacted]	[redacted]	[redacted]
hCOCT	14-HCOCT	sn	*M*	*30*	0	91	93	136	69	126	84	54	115	[redacted]	[redacted]	[redacted]	[redacted]	[redacted]	[redacted]	[redacted]	[redacted]	[redacted]
hCOCT	15-HCOCT	dx	*M*	*55*	0	101	97	141	64	141	143	91	101	[redacted]	[redacted]	[redacted]	[redacted]	[redacted]	[redacted]	[redacted]	[redacted]	[redacted]
hCOCT	15-HCOCT	sn	*M*	*55*	0	101	97	138	60	124	112	81	93	[redacted]	[redacted]	[redacted]	[redacted]	[redacted]	[redacted]	[redacted]	[redacted]	[redacted]
hCOCT	16-HCOCT	dx	*F*	*30*	0	103	103	154	84	146	107	86	84	[redacted]	[redacted]	[redacted]	[redacted]	[redacted]	[redacted]	[redacted]	[redacted]	[redacted]
hCOCT	16-HCOCT	sn	*F*	*30*	0	103	103	165	73	147	99	77	104	[redacted]	[redacted]	[redacted]	[redacted]	[redacted]	[redacted]	[redacted]	[redacted]	[redacted]
hCOCT	17-HCOCT	dx	*F*	*35*	0	90	91	134	66	133	104	64	96	[redacted]	[redacted]	[redacted]	[redacted]	[redacted]	[redacted]	[redacted]	[redacted]	[redacted]
hCOCT	17-HCOCT	sn	*F*	*35*	0	90	91	139	64	129	101	67	96	[redacted]	[redacted]	[redacted]	[redacted]	[redacted]	[redacted]	[redacted]	[redacted]	[redacted]
hCOCT	18-HCOCT	dx	*F*	*35*	0	98	93	138	70	134	117	62	113	[redacted]	[redacted]	[redacted]	[redacted]	[redacted]	[redacted]	[redacted]	[redacted]	[redacted]
hCOCT	18-HCOCT	sn	*F*	*35*	0	98	93	156	69	133	97	63	106	[redacted]	[redacted]	[redacted]	[redacted]	[redacted]	[redacted]	[redacted]	[redacted]	[redacted]
hCOCT	19-HCOCT	dx	*M*	*32*	0	105	105	169	89	134	125	88	100	[redacted]	[redacted]	[redacted]	[redacted]	[redacted]	[redacted]	[redacted]	[redacted]	[redacted]
hCOCT	19-HCOCT	sn	*M*	*32*	0	105	105	156	77	128	127	79	75	[redacted]	[redacted]	[redacted]	[redacted]	[redacted]	[redacted]	[redacted]	[redacted]	[redacted]
hCOCT	20-HCOCT	dx	*M*	*33*	0	115	121	142	63	142	149	61	147	[redacted]	[redacted]	[redacted]	[redacted]	[redacted]	[redacted]	[redacted]	[redacted]	[redacted]
hCOCT	20-HCOCT	sn	*M*	*33*	0	115	121	176	78	170	175	111	160	[redacted]	[redacted]	[redacted]	[redacted]	[redacted]	[redacted]	[redacted]	[redacted]	[redacted]

S3 Table. OCT and MRI parameters of the study population *(continue)*.

group	Code	OR	gender	age	ON (1= yes)	LE	RE	T-ipsilat.			N-controlat			WM			ipsilateral (to T-RNFL) ORWM				CTh	
						Gsn	Gdx	TI	T	TS	NI	N	NS	WMLV	WMV	%WMLV	OR WMLV mm^3	ORV	ORWMLV fraction (%)	ratio/ ratio	pericalcarine	global
hCOCT	21-HCOCT	dx	*M*	*25*	0	107	105	178	85	130	114	61	118									
hCOCT	21-HCOCT	sn	*M*	*25*	0	107	105	187	84	150	109	74	106									
hCOCT	22-HCOCT	dx	*F*	*45*	0	89	91	116	65	120	100	65	116									
hCOCT	22-HCOCT	sn	*F*	*45*	0	89	91	127	61	124	134	66	101									
hCOCT	23-HCOCT	dx	*F*	*34*	0	103	107	175	87	155	83	71	130									
hCOCT	23-HCOCT	sn	*F*	*34*	0	103	107	159	75	168	87	75	114									
hCOCT	24-HCOCT	dx	*M*	*59*	0	83	86	122	64	122	77	55	110									
hCOCT	24-HCOCT	sn	*M*	*59*	0	83	86	126	61	121	79	59	118									
hCOCT	25-HCOCT	dx	*F*	*26*	0	98	94	146	84	126	89	55	110									
hCOCT	25-HCOCT	sn	*F*	*26*	0	98	94	154	94	130	88	61	101									
hCOCT	26-HCOCT	dx	*F*	*28*	0	117	116	171	79	166	149	85	143									
hCOCT	26-HCOCT	sn	*F*	*28*	0	117	116	188	73	138	150	79	125									
hCOCT	27-HCOCT	dx	*F*	*27*	0	97	97	144	86	138	88	73	109									
hCOCT	27-HCOCT	sn	*F*	*27*	0	97	97	151	76	133	92	66	97									
hCOCT	28-HCOCT	dx	*F*	*31*	0	101	102	134	68	124	129	91	133									
hCOCT	28-HCOCT	sn	*F*	*31*	0	101	102	121	63	116	128	99	97									
hCOCT	29-HCOCT	dx	*M*	*28*	0	98	96	145	72	139	126	65	127									
hCOCT	29-HCOCT	sn	*M*	*28*	0	98	96	140	68	134	106	64	108									
hCOCT	30-HCOCT	dx	*M*	*48*	0	83	82	127	69	125	90	48	82									
hCOCT	30-HCOCT	sn	*M*	*48*	0	83	82	138	67	122	87	52	75									

S3 Table. OCT and MRI parameters of the study population *(continue)*.

						LE	RE	T-ipsilat.			N-controlat			WM			ipsilateral (to T-RNFL) ORWM				CTh	
group	Code	OR	gender	age	ON (1= yes)	Gsn	Gdx	TI	T	TS	NI	N	NS	WMLV	WMV	%WMLV	OR WMLV mm^3	ORV	ORWMLV fraction (%)	ratio/ ratio	pericalcarine	global
hCOCT	31-HCOCT	dx	*F*	*32*	0	112	108	173	81	156	152	79	121									
hCOCT	31-HCOCT	sn	*F*	*32*	0	112	108	162	68	168	117	62	130									
hCRM	01-HCMRI	dx	*F*	*48*	0																1,99	2,55
hCRM	02-HCMRI	dx	*F*	*18*	0																1,68	2,37
hCRM	03-HCMRI	dx	*F*	*39*	0																1,52	2,31
hCRM	04-HCMRI	dx	*F*	*44*	0																1,86	2,37
hCRM	05-HCMRI	dx	*F*	*19*	0																1,77	2,47
hCRM	06-HCMRI	dx	*F*	*42*	0																1,70	2,58
hCRM	07-HCMRI	dx	*F*	*23*	0																1,92	2,60
hCRM	08-HCMRI	dx	*F*	*48*	0																1,76	2,44
hCRM	09-HCMRI	dx	*M*	*42*	0																1,64	2,30
hCRM	10-HCMRI	dx	*M*	*22*	0																1,49	2,33
hCRM	11-HCMRI	dx	*F*	*62*	0																1,99	2,60
hCRM	12-HCMRI	dx	*F*	*45*	0																1,69	2,46
hCRM	13-HCMRI	dx	*F*	*22*	0																1,94	2,46
hCRM	14-HCMRI	dx	*M*	*41*	0																1,67	2,57
hCRM	15-HCMRI	dx	*F*	*18*	0																1,77	2,51
hCRM	16-HCMRI	dx	*F*	*50*	0																1,56	2,54
hCRM	17-HCMRI	dx	*F*	*36*	0																1,43	2,26
hCRM	18-HCMRI	dx	*F*	*18*	0																1,65	2,49

S3 Table. OCT and MRI parameters of the study population *(continue)*.

group	Code	OR	gender	age	ON (1= yes)	LE	RE	T-ipsilat.			N-controlat			WM			ipsilateral (to T-RNFL) ORWM				CTh	
						Gsn	Gdx	TI	T	TS	NI	N	NS	WMLV	WMV	%WMLV	OR WMLV mm^3	ORV	ORWMLV fraction (%)	ratio/ ratio	pericalcarine	global
hCRM	19-HCMRI	dx	*M*	*19*	0	[redacted]	[redacted]	[redacted]	[redacted]	[redacted]	[redacted]	[redacted]	[redacted]	[redacted]	[redacted]	[redacted]	[redacted]	[redacted]	[redacted]	[redacted]	1,95	2,73
hCRM	20-HCMRI	dx	*F*	*31*	0	[redacted]	[redacted]	[redacted]	[redacted]	[redacted]	[redacted]	[redacted]	[redacted]	[redacted]	[redacted]	[redacted]	[redacted]	[redacted]	[redacted]	[redacted]	1,50	2,40
hCRM	21-HCMRI	dx	*M*	*66*	0	[redacted]	[redacted]	[redacted]	[redacted]	[redacted]	[redacted]	[redacted]	[redacted]	[redacted]	[redacted]	[redacted]	[redacted]	[redacted]	[redacted]	[redacted]	1,80	2,40
hCRM	22-HCMRI	dx	*F*	*53*	0	[redacted]	[redacted]	[redacted]	[redacted]	[redacted]	[redacted]	[redacted]	[redacted]	[redacted]	[redacted]	[redacted]	[redacted]	[redacted]	[redacted]	[redacted]	1,74	2,49
hCRM	23-HCMRI	dx	*F*	*30*	0	[redacted]	[redacted]	[redacted]	[redacted]	[redacted]	[redacted]	[redacted]	[redacted]	[redacted]	[redacted]	[redacted]	[redacted]	[redacted]	[redacted]	[redacted]	2,06	2,47
hCRM	24-HCMRI	dx	*F*	*47*	0	[redacted]	[redacted]	[redacted]	[redacted]	[redacted]	[redacted]	[redacted]	[redacted]	[redacted]	[redacted]	[redacted]	[redacted]	[redacted]	[redacted]	[redacted]	1,88	2,43
hCRM	25-HCMRI	dx	*M*	*46*	0	[redacted]	[redacted]	[redacted]	[redacted]	[redacted]	[redacted]	[redacted]	[redacted]	[redacted]	[redacted]	[redacted]	[redacted]	[redacted]	[redacted]	[redacted]	1,98	2,65
hCRM	26-HCMRI	dx	*F*	*18*	0	[redacted]	[redacted]	[redacted]	[redacted]	[redacted]	[redacted]	[redacted]	[redacted]	[redacted]	[redacted]	[redacted]	[redacted]	[redacted]	[redacted]	[redacted]	1,84	2,61
hCRM	27-HCMRI	dx	*F*	*27*	0	[redacted]	[redacted]	[redacted]	[redacted]	[redacted]	[redacted]	[redacted]	[redacted]	[redacted]	[redacted]	[redacted]	[redacted]	[redacted]	[redacted]	[redacted]	1,63	2,37
hCRM	28-HCMRI	dx	*F*	*37*	0	[redacted]	[redacted]	[redacted]	[redacted]	[redacted]	[redacted]	[redacted]	[redacted]	[redacted]	[redacted]	[redacted]	[redacted]	[redacted]	[redacted]	[redacted]	1,78	2,44
hCRM	01-HCMRI	sn	*F*	*48*	0	[redacted]	[redacted]	[redacted]	[redacted]	[redacted]	[redacted]	[redacted]	[redacted]	[redacted]	[redacted]	[redacted]	[redacted]	[redacted]	[redacted]	[redacted]	1,84	2,55
hCRM	02-HCMRI	sn	*F*	*18*	0	[redacted]	[redacted]	[redacted]	[redacted]	[redacted]	[redacted]	[redacted]	[redacted]	[redacted]	[redacted]	[redacted]	[redacted]	[redacted]	[redacted]	[redacted]	1,49	2,37
hCRM	03-HCMRI	sn	*F*	*39*	0	[redacted]	[redacted]	[redacted]	[redacted]	[redacted]	[redacted]	[redacted]	[redacted]	[redacted]	[redacted]	[redacted]	[redacted]	[redacted]	[redacted]	[redacted]	1,68	2,31
hCRM	04-HCMRI	sn	*F*	*44*	0	[redacted]	[redacted]	[redacted]	[redacted]	[redacted]	[redacted]	[redacted]	[redacted]	[redacted]	[redacted]	[redacted]	[redacted]	[redacted]	[redacted]	[redacted]	1,78	2,37
hCRM	05-HCMRI	sn	*F*	*19*	0	[redacted]	[redacted]	[redacted]	[redacted]	[redacted]	[redacted]	[redacted]	[redacted]	[redacted]	[redacted]	[redacted]	[redacted]	[redacted]	[redacted]	[redacted]	1,97	2,47
hCRM	06-HCMRI	sn	*F*	*42*	0	[redacted]	[redacted]	[redacted]	[redacted]	[redacted]	[redacted]	[redacted]	[redacted]	[redacted]	[redacted]	[redacted]	[redacted]	[redacted]	[redacted]	[redacted]	1,81	2,58
hCRM	07-HCMRI	sn	*F*	*23*	0	[redacted]	[redacted]	[redacted]	[redacted]	[redacted]	[redacted]	[redacted]	[redacted]	[redacted]	[redacted]	[redacted]	[redacted]	[redacted]	[redacted]	[redacted]	2,10	2,60
hCRM	08-HCMRI	sn	*F*	*48*	0	[redacted]	[redacted]	[redacted]	[redacted]	[redacted]	[redacted]	[redacted]	[redacted]	[redacted]	[redacted]	[redacted]	[redacted]	[redacted]	[redacted]	[redacted]	1,57	2,44
hCRM	09-HCMRI	sn	*M*	*42*	0	[redacted]	[redacted]	[redacted]	[redacted]	[redacted]	[redacted]	[redacted]	[redacted]	[redacted]	[redacted]	[redacted]	[redacted]	[redacted]	[redacted]	[redacted]	1,46	2,30
hCRM	10-HCMRI	sn	*M*	*22*	0	[redacted]	[redacted]	[redacted]	[redacted]	[redacted]	[redacted]	[redacted]	[redacted]	[redacted]	[redacted]	[redacted]	[redacted]	[redacted]	[redacted]	[redacted]	1,38	2,33

S3 Table. OCT and MRI parameters of the study population *(continue)*.

group	<u>Code</u>	OR	gender	age	ON (1= yes)	LE	RE	T-ipsilat.			N-controlat			WM			ipsilateral (to T-RNFL) ORWM				CTh	
						Gsn	Gdx	TI	T	TS	NI	N	NS	WMLV	WMV	%WMLV	OR WMLV mm^3	ORV	ORWMLV fraction (%)	ratio/ ratio	pericalcarine	global
hCRM	11-HCMRI	sn	*F*	*62*	0																2,09	2,60
hCRM	12-HCMRI	sn	*F*	*45*	0																1,65	2,46
hCRM	13-HCMRI	sn	*F*	*22*	0																1,93	2,46
hCRM	14-HCMRI	sn	*M*	*41*	0																1,61	2,57
hCRM	15-HCMRI	sn	*F*	*18*	0																1,70	2,51
hCRM	16-HCMRI	sn	*F*	*50*	0																1,63	2,54
hCRM	17-HCMRI	sn	*F*	*36*	0																1,48	2,26
hCRM	18-HCMRI	sn	*F*	*18*	0																1,66	2,49
hCRM	19-HCMRI	sn	*M*	*19*	0																1,86	2,73
hCRM	20-HCMRI	sn	*F*	*31*	0																1,38	2,40
hCRM	21-HCMRI	sn	*M*	*66*	0																1,86	2,40
hCRM	22-HCMRI	sn	*F*	*53*	0																1,75	2,49
hCRM	23-HCMRI	sn	*F*	*30*	0																2,22	2,47
hCRM	24-HCMRI	sn	*F*	*47*	0																1,99	2,43
hCRM	25-HCMRI	sn	*M*	*46*	0																2,02	2,65
hCRM	26-HCMRI	sn	*F*	*18*	0																1,86	2,61
hCRM	27-HCMRI	sn	*F*	*27*	0																1,46	2,37
hCRM	28-HCMRI	sn	*F*	*37*	0																1,96	2,44
NOR-	25-CIS/eRRMS	dx	*F*	*34*	0	96	97	172	85	124	121	60	94	19,5	463498,9	0,00%	0,0	25011,18	0,00%	0,0	1,57	2,47
NOR-	11-CIS/eRRMS	dx	*M*	*27*	0	105	106	190	75	120	133	78	131	1181,0	577625,3	0,20%	22,2	31814,54	0,07%	0,3	1,65	2,24

S3 Table. OCT and MRI parameters of the study population *(continue)*.

group	Code	OR	gender	age	ON (1= yes)	LE	RE	T-ipsilat.			N-controlat			WM			ipsilateral (to T-RNFL) ORWM				CTh	
						Gsn	Gdx	TI	T	TS	NI	N	NS	WMLV	WMV	%WMLV	OR WMLV mm^3	ORV	ORWMLV fraction (%)	ratio/ ratio	pericalcarine	global
NOR-	19-CIS/eRRMS	dx	F	22	0	93	104	175	101	141	117	53	70	3796,5	463375,5	0,82%	348,1	25053,59	1,39%	1,7	1,45	2,41
NOR-	04-CIS/eRRMS	dx	F	30	0	90	94	148	112	123	76	52	90	2662,3	443001,6	0,60%	192,9	23718,38	0,81%	1,4	1,70	2,43
NOR-	30-CIS/eRRMS	dx	F	49	0	110	109	159	86	166	131	85	128	2097,1	520069,9	0,40%	274,5	28031,09	0,98%	2,4	1,87	2,48
NOR-	27-CIS/eRRMS	dx	F	42	0	104	105	163	89	151	107	88	110	1265,3	401049,7	0,32%	125,7	23980,84	0,52%	1,7	1,51	2,43
NOR-	31-CIS/eRRMS	dx	M	21	0	95	90	152	60	116	118	78	130	703,8	460451,4	0,15%	60,4	25334,8	0,24%	0,92	1,65	2,83
NOR-	28-CIS/eRRMS	dx	F	59	0	104	107	173	79	166	152	75	135	2176,8	448975,3	0,48%	441,7	23993,08	1,84%	3,8	1,63	2,40
NOR-	12-CIS/eRRMS	dx	F	33	0	98	97	165	88	122	83	74	98	5381,7	484919,5	1,11%	261,8	27749,81	0,94%	0,9	1,37	2,39
NOR-	18-CIS/eRRMS	dx	F	27	0	107	109	173	77	177	125	102	136	2584,9	426636,3	0,61%	20,4	30220,9	0,07%	0,1	2,00	2,55
NOR-	39-CIS/eRRMS	dx	F	45	0	98	98	161	68	144	106	81	109	1297,2	553935,1	0,23%	0,0	29481,1	0,00%	0,11	1,62	2,38
NOR-	33-CIS/eRRMS	dx	F	23	0	87	82	143	73	118	87	65	86	586,9	466703,4	0,13%	38,7	25074,6	0,15%	0,76	1,78	2,52
NOR-	13-CIS/eRRMS	dx	M	21	0	97	97	160	78	127	121	83	90	349,3	568443,4	0,06%	7,0	31103,02	0,02%	0,4	2,14	2,44
NOR-	14-CIS/eRRMS	dx	M	44	0	106	100	142	66	153	123	83	169	129,5	552205,1	0,02%	0,0	30304,77	0,00%	0,0	1,88	2,42
NOR-	05-CIS/eRRMS	dx	M	47	0	79	83	145	76	113	72	39	75	4596,0	428620,1	1,07%	182,0	29627,23	0,61%	0,6	1,46	2,27
NOR-	09-CIS/eRRMS	dx	M	42	0	96	98	134	77	132	129	91	99	1334,7	468711,1	0,28%	47,9	34564,97	0,14%	0,5	1,56	2,51
NOR-	32-CIS/eRRMS	dx	M	43	0	110	116	168	77	177	137	76	123	1403,9	548057,5	0,26%	25,9	31845,5	0,08%	0,16	1,60	2,40
NOR-	34-CIS/eRRMS	dx	F	42	0	102	104	162	74	142	126	84	120	945,1	468121,1	0,20%	146,2	25285,4	0,58%	1,41	1,59	2,43
NOR-	06-CIS/eRRMS	dx	F	25	0	118	119	144	87	143	175	88	110	592,9	572330,1	0,10%	99,5	28108,38	0,35%	3,4	1,53	2,39
NOR-	38-CIS/eRRMS	dx	F	34	0	108	110	150	82	135	138	90	95	222,5	430901,5	0,05%	0,0	23743,6	0,00%	0,19	1,72	2,56
NOR-	07-CIS/eRRMS	dx	M	24	0	88	86	121	64	124	121	64	81	8768,5	401851,0	2,18%	323,0	25425,87	1,27%	0,6	1,80	2,46
NOR-	01-CIS/eRRMS	dx	F	37	0	105	96	151	76	136	105	115	178	4220,7	467965,3	0,90%	152,0	19086,39	0,80%	0,9	1,68	2,57

S3 Table. OCT and MRI parameters of the study population *(continue)*.

group	Code	OR	gender	age	ON (1= yes)	LE	RE	T-ipsilat.			N-controlat			WM			ipsilateral (to T-RNFL) ORWM				CTh	
						Gsn	Gdx	TI	T	TS	NI	N	NS	WMLV	WMV	%WMLV	OR WMLV mm^3	ORV	ORWMLV fraction (%)	ratio/ ratio	pericalcarine	global
NOR-	21-CIS/eRRMS	dx	*F*	*39*	0	88	89	132	73	127	69	77	95	314,3	482063,2	0,07%	0,0	26102,47	0,00%	0,0	1,54	2,33
NOR-	02-CIS/eRRMS	dx	F	50	0	99	96	158	68	146	140	80	113	2216,9	404351,2	0,55%	51,1	23209,19	0,22%	0,4	1,73	2,42
NOR-	03-CIS/eRRMS	dx	F	47	0	84	83	151	71	104	119	64	67	1829,1	433968,5	0,42%	263,2	25632,41	1,03%	2,4	2,20	2,62
NOR-	15-CIS/eRRMS	dx	*M*	*30*	0	104	108	133	61	156	153	69	129	1490,0	472054,5	0,32%	0,0	24981,31	0,00%	0,0	1,94	2,34
NOR-	10-CIS/eRRMS	dx	M	26	0	92	84	112	55	115	94	117	98	6797,8	522182,4	1,30%	560,3	28751,61	1,95%	1,5	1,56	2,51
NOR-	24-CIS/eRRMS	dx	*F*	*32*	0	105	108	175	71	165	129	63	139	1949,0	470712,9	0,41%	61,0	24926,24	0,24%	0,6	1,52	2,30
NOR-	35-CIS/eRRMS	dx	*F*	18	0	105	101	122	64	132	150	95	122	174,4	369487,6	0,05%	0,0	23271,5	0,00%	0,12	1,60	2,67
NOR-	22-CIS/eRRMS	dx	*M*	*42*	0	96	98	137	63	120	105	75	124	1282,4	528226,4	0,24%	93,3	28229,56	0,33%	1,4	1,71	2,56
NOR-	23-CIS/eRRMS	dx	*M*	*18*	0	97	94	121	71	157	118	93	108	2712,1	420164,3	0,65%	646,0	25898,19	2,49%	3,9	1,83	2,61
NOR-	16-CIS/eRRMS	dx	*M*	*26*	0	105	111	164	70	136	148	101	132	2920,4	630583,3	0,46%	0,0	34933,45	0,00%	0,0	1,89	2,57
NOR-	40-CIS/eRRMS	dx	*F*	40	0	112	111	176	76	146	155	86	123	5,8	495859,0	0,00%	0,0	25754,2	0,00%	0	1,66	2,56
NOR-	20-CIS/eRRMS	dx	*F*	*28*	0	98	89	90	57	154	137	58	147	6051,2	473201,4	1,28%	56,7	25743,2	0,22%	0,2	1,64	2,34
NOR-	36-CIS/eRRMS	dx	*F*	21	0	90	85	125	67	109	90	73	112	713,4	517308,4	0,14%	104,6	27395,8	0,38%	2,88	1,93	2,67
NOR-	08-CIS/eRRMS	dx	F	35	0	101	102	147	78	132	122	60	117	693,1	469508,6	0,15%	5,0	23434,06	0,02%	0,1	1,77	2,50
NOR-	26-CIS/eRRMS	dx	*F*	*46*	0	98	97	130	71	135	148	71	112	918,0	643061,8	0,14%	48,9	29525,64	0,17%	1,2	1,71	2,39
NOR-	17-CIS/eRRMS	dx	M	46	0	113	110	170	81	159	126	93	131	1262,3	437644,7	0,29%	20,0	24587,69	0,08%	0,3	1,57	2,46
NOR-	37-CIS/eRRMS	dx	*F*	24	0	110	109	157	72	146	154	105	139	18050,9	500142,0	3,61%	349,9	27167,4	1,29%	1,34	1,76	2,60
NOR-	29-CIS/eRRMS	dx	*F*	23	0	106	101	134	63	137	147	88	147	4065,1	515217,4	0,79%	213,3	27083,76	0,79%	1,0	1,84	2,61
NOR-	25-CIS/eRRMS	sn	*F*	*34*	0	96	97	167	75	117	102	55	97	19,5	463498,9	0,00%	0,0	23957,03	0,00%	0,0	1,83	2,47
NOR-	11-CIS/eRRMS	sn	*M*	*27*	0	105	106	170	68	115	128	79	104	1181,0	577625,3	0,20%	136,0	30473,64	0,45%	2,2	1,68	2,24

S3 Table. OCT and MRI parameters of the study population *(continue)*.

						LE	RE	T-ipsilat.			N-controlat			WM			ipsilateral (to T-RNFL) ORWM				CTh	
group	Code	OR	gender	age	ON (1= yes)	Gsn	Gdx	TI	T	TS	NI	N	NS	WMLV	WMV	%WMLV	OR WMLV mm^3	ORV	ORWMLV fraction (%)	ratio/ ratio	pericalcarine	global
NOR-	19-CIS/eRRMS	sn	F	22	0	93	104	166	76	135	106	63	83	3796,5	463375,5	0,82%	636,1	26177,59	2,43%	3,0	1,48	2,41
NOR-	04-CIS/eRRMS	sn	F	30	0	90	94	150	85	132	76	52	74	2662,3	443001,6	0,60%	143,8	24790,88	0,58%	1,0	1,66	2,43
NOR-	30-CIS/eRRMS	sn	*F*	49	0	110	109	149	79	146	105	70	131	2097,1	520069,9	0,40%	88,9	29264,5	0,30%	0,8	1,92	2,48
NOR-	27-CIS/eRRMS	sn	*F*	*42*	0	104	105	153	67	154	94	80	98	1265,3	401049,7	0,32%	216,6	22970,12	0,94%	3,0	1,62	2,43
NOR-	31-CIS/eRRMS	sn	M	21	0	95	90	139	54	109	105	65	95	703,8	460451,4	0,15%	12,1	26449,6	0,05%	0,92	1,65	2,83
NOR-	28-CIS/eRRMS	sn	*F*	*59*	0	104	107	154	57	127	108	58	134	2176,8	448975,3	0,48%	82,1	25048,82	0,33%	0,7	1,72	2,40
NOR-	12-CIS/eRRMS	sn	*F*	*33*	0	98	97	160	76	142	93	67	84	5381,7	484919,5	1,11%	895,8	26580,24	3,37%	3,0	1,40	2,39
NOR-	18-CIS/eRRMS	sn	F	27	0	107	109	151	55	128	100	73	121	2584,9	426636,3	0,61%	73,8	31550,67	0,23%	0,4	1,92	2,55
NOR-	39-CIS/eRRMS	sn	*F*	45	0	98	98	142	61	145	95	80	87	1297,2	553935,1	0,23%	15,7	30778,3	0,05%	0,11	1,56	2,38
NOR-	33-CIS/eRRMS	sn	*F*	23	0	87	82	144	67	118	80	47	74	586,9	466703,4	0,13%	10,6	26178,0	0,04%	0,76	1,77	2,52
NOR-	13-CIS/eRRMS	sn	M	21	0	97	97	139	65	131	95	71	95	349,3	568443,4	0,06%	38,9	32504,42	0,12%	1,9	2,01	2,44
NOR-	14-CIS/eRRMS	sn	*M*	*44*	0	106	100	118	62	152	114	72	112	129,5	552205,1	0,02%	88,8	29027,51	0,31%	13,0	1,77	2,42
NOR-	05-CIS/eRRMS	sn	M	47	0	79	83	131	76	125	73	49	83	4596,0	428620,1	1,07%	81,1	30954,96	0,26%	0,2	1,45	2,27
NOR-	09-CIS/eRRMS	sn	M	42	0	96	98	120	62	118	114	82	89	1334,7	468711,1	0,28%	171,2	36138,3	0,47%	1,7	1,63	2,51
NOR-	32-CIS/eRRMS	sn	M	43	0	110	116	163	73	159	123	79	146	1403,9	548057,5	0,26%	0,9	33246,7	0,00%	0,16	1,71	2,40
NOR-	34-CIS/eRRMS	sn	*F*	42	0	102	104	141	66	131	106	76	122	945,1	468121,1	0,20%	1,4	26398,0	0,01%	1,41	1,55	2,43
NOR-	06-CIS/eRRMS	sn	F	25	0	118	119	183	76	152	203	87	112	592,9	572330,1	0,10%	63,9	29359,47	0,22%	2,1	1,58	2,39
NOR-	38-CIS/eRRMS	sn	*F*	34	0	108	110	176	71	132	165	83	103	222,5	430901,5	0,05%	4,7	24788,3	0,02%	0,19	1,81	2,56
NOR-	07-CIS/eRRMS	sn	M	24	0	88	86	136	57	126	100	62	86	8768,5	401851,0	2,18%	820,5	26555,39	3,09%	1,4	1,75	2,46
NOR-	01-CIS/eRRMS	sn	F	37	0	105	96	114	47	117	62	67	132	4220,7	467965,3	0,90%	74,9	19941,25	0,38%	0,4	1,58	2,57

S3 Table. OCT and MRI parameters of the study population *(continue)*.

group	Code	OR	gender	age	ON (1= yes)	LE	RE	T-ipsilat.			N-controlat			WM			ipsilateral (to T-RNFL) ORWM				CTh	
						Gsn	Gdx	TI	T	TS	NI	N	NS	WMLV	WMV	%WMLV	OR WMLV mm^3	ORV	ORWMLV fraction (%)	ratio/ ratio	pericalcarine	global
NOR-	21-CIS/eRRMS	sn	*F*	*39*	0	88	89	124	65	130	79	76	82	314,3	482063,2	0,07%	0,0	25002,32	0,00%	0,0	1,57	2,33
NOR-	02-CIS/eRRMS	sn	F	50	0	99	96	142	57	121	110	59	102	2216,9	404351,2	0,55%	57,9	24266,7	0,24%	0,4	1,68	2,42
NOR-	03-CIS/eRRMS	sn	F	47	0	84	83	141	57	100	90	54	67	1829,1	433968,5	0,42%	301,5	26766,66	1,13%	2,7	2,15	2,62
NOR-	15-CIS/eRRMS	sn	*M*	*30*	0	104	108	146	61	146	156	86	127	1490,0	472054,5	0,32%	0,0	23928,42	0,00%	0,0	1,73	2,34
NOR-	10-CIS/eRRMS	sn	M	26	0	92	84	102	51	107	101	72	93	6797,8	522182,4	1,30%	369,5	30059,1	1,23%	0,9	1,40	2,51
NOR-	24-CIS/eRRMS	sn	*F*	*32*	0	105	108	156	69	148	125	70	120	1949,0	470712,9	0,41%	41,5	23875,67	0,17%	0,4	1,58	2,30
NOR-	35-CIS/eRRMS	sn	*F*	18	0	105	101	131	60	123	151	83	110	174,4	369487,6	0,05%	2,6	24295,5	0,01%	0,12	1,58	2,67
NOR-	22-CIS/eRRMS	sn	*M*	*42*	0	96	98	145	66	113	123	89	98	1282,4	528226,4	0,24%	73,6	27039,76	0,27%	1,1	1,88	2,56
NOR-	23-CIS/eRRMS	sn	*M*	*18*	0	97	94	147	54	113	88	69	105	2712,1	420164,3	0,65%	578,5	24806,65	2,33%	3,6	1,68	2,61
NOR-	16-CIS/eRRMS	sn	*M*	*26*	0	105	111	153	54	96	156	89	113	2920,4	630583,3	0,46%	1146,0	36470,58	3,14%	6,8	2,06	2,57
NOR-	40-CIS/eRRMS	sn	*F*	40	0	112	111	167	67	146	130	75	132	5,8	495859,0	0,00%	0,0	26887,4	0,00%	0	1,65	2,56
NOR-	20-CIS/eRRMS	sn	*F*	*28*	0	98	89	118	57	149	103	60	130	6051,2	473201,4	1,28%	316,9	24658,2	1,29%	1,0	1,56	2,34
NOR-	36-CIS/eRRMS	sn	*F*	21	0	90	85	135	62	112	81	66	94	713,4	517308,4	0,14%	117,6	28601,2	0,41%	2,88	2,05	2,67
NOR-	08-CIS/eRRMS	sn	F	35	0	101	102	140	83	141	127	70	111	693,1	469508,6	0,15%	294,0	24482,58	1,20%	8,1	1,65	2,50
NOR-	26-CIS/eRRMS	sn	*F*	*46*	0	98	97	109	61	148	131	74	88	918,0	643061,8	0,14%	0,0	30824,81	0,00%	0,0	1,64	2,39
NOR-	17-CIS/eRRMS	sn	M	46	0	113	110	166	72	151	107	91	101	1262,3	437644,7	0,29%	78,1	25698,7	0,30%	1,1	1,80	2,46
NOR-	37-CIS/eRRMS	sn	*F*	24	0	110	109	128	57	137	151	82	111	18050,9	500142,0	3,61%	2337,5	28362,8	8,24%	1,34	1,80	2,60
NOR-	29-CIS/eRRMS	sn	*F*	*23*	0	106	101	123	58	138	139	79	116	4065,1	515217,4	0,79%	242,4	25942,26	0,93%	1,2	1,77	2,61
NOR+	43-CIS/eRRMS	dx	M	56	1	79	61	39	23	79	121	75	99	10716,7	464668,1	0,023	555,4	26973,25	0,02	0,9	1,56	2,11
NOR+	48-CIS/eRRMS	dx	M	23	0	91	86	117	54	135	113	77	130	15984,2	462521,5	0,035	1348,1	27201,46	0,05	1,4	1,59	2,56

S3 Table. OCT and MRI parameters of the study population *(continue)*.

group	Code	OR	gender	age	ON (1= yes)	LE	RE	T-ipsilat.			N-controlat			WM			ipsilateral (to T-RNFL) ORWM			ratio/ ratio	CTh	
						Gsn	Gdx	TI	T	TS	NI	N	NS	WMLV	WMV	%WMLV	OR WMLV mm^3	ORV	ORWMLV fraction (%)		pericalcarine	global
NOR+	46-CIS/eRRMS	dx	F	29	0	109	116	153	75	171	150	94	140	1929,2	516296,5	0,004	10,8	25613,23	0,00	0,1	1,65	2,39
NOR+	50-CIS/eRRMS	dx	M	32	0	43	105	142	88	157	51	39	55	13133,6	561946,7	0,023	2305,1	28188,71	0,08	3,5	1,45	2,46
NOR+	47-CIS/eRRMS	dx	F	44	0	47	108	167	93	133	68	42	61	2900,7	474520,4	0,006	55,0	25809,13	0,00	0,3	1,57	2,29
NOR+	42-CIS/eRRMS	dx	F	39	1	90	81	112	49	109	79	58	115	1513,6	511968,0	0,003	89,5	30553,62	0,00	1,0	1,62	2,39
NOR+	49-CIS/eRRMS	dx	F	25	0	102	105	133	69	186	157	72	147	5,6	493332,4	0,000	0,0	24852,05	0,00	0,0	1,49	2,36
NOR+	44-CIS/eRRMS	dx	M	42	1	128	116	177	81	192	182	119	140	1087,4	529045,1	0,002	35,5	28112,43	0,00	0,6	1,77	2,37
NOR+	45-CIS/eRRMS	dx	F	22	0	97	83	145	69	108	139	67	111	457,3	453158,8	0,001	41,0	24357,54	0,00	1,7	1,88	2,45
NOR+	43-CIS/eRRMS	sn	M	56	0	79	61	97	31	105	102	66	90	10716,7	464668,1	0,023	2229,5	25836,4	0,09	3,7	1,86	2,11
NOR+	48-CIS/eRRMS	sn	M	23	1	91	86	120	46	123	87	65	114	15984,2	462521,5	0,035	1164,1	28398,37	0,04	1,2	1,98	2,56
NOR+	46-CIS/eRRMS	sn	F	29	1	109	116	121	58	154	135	93	132	1929,2	516296,5	0,004	0,0	26740,25	0,00	0,0	1,82	2,39
NOR+	50-CIS/eRRMS	sn	M	32	1	43	105	54	28	50	106	63	131	13133,6	561946,7	0,023	590,9	29429,06	0,02	0,9	1,53	2,46
NOR+	47-CIS/eRRMS	sn	F	44	1	47	104	58	24	62	125	78	99	2900,7	474520,4	0,006	540,3	26944,77	0,02	3,3	1,69	2,29
NOR+	42-CIS/eRRMS	sn	F	39	0	90	81	133	67	142	77	72	109	1513,6	511968,0	0,003	290,7	29265,87	0,01	3,4	1,56	2,39
NOR+	49-CIS/eRRMS	sn	F	25	1	102	105	107	57	144	105	63	154	5,6	493332,4	0,000	0,0	25945,59	0,00	0,0	1,62	2,36
NOR+	44-CIS/eRRMS	sn	M	42	0	128	116	178	66	153	121	69	136	1087,4	529045,1	0,002	16,6	26927,57	0,00	0,3	1,85	2,37
NOR+	41-CIS/eRRMS	sn	F	34	0	97	86	110	47	104	129	69	114	599,4	445724,0	0,001	170,9	24345,64	0,01	5,2	1,56	2,59
NOR+	45-CIS/eRRMS	sn	F	22	1	97	83	149	57	129	119	49	57	457,3	453158,8	0,001	0,7	25429,31	0,00	0,0	1,93	2,45
NOR+	41-CIS/eRRMS	dx	F	34	1	97	86	116	41	112	127	99	140	599,4	445724,0	0,001	6,7	25437,83	0,00	0,2	1,33	2,59
